# Memory for fearful faces across development: specialization of amygdala nuclei and medial temporal lobe structures

**DOI:** 10.3389/fnhum.2013.00901

**Published:** 2013-12-25

**Authors:** Charlotte Pinabiaux, Lucie Hertz-Pannier, Catherine Chiron, Sébastian Rodrigo, Isabelle Jambaqué, Marion Noulhiane

**Affiliations:** ^1^U663, Neuropediatric Department, Inserm, University Paris Descartes, Paris and CEASaclay, France; ^2^UNIACT, NeuroSpin, I2BM, DSV, CEAGif Sur Yvette, France; ^3^Psychology Department, PRES Paris Sorbonne, Paris Descartes UniversityBoulogne-Billancourt, France; ^4^Neuropediatric Department, Necker HospitalParis, France

**Keywords:** emotional modulation of memory, fearful faces, medial temporal lobe, amygdaloid complex, children, adolescents

## Abstract

Enhanced memory for emotional faces is a significant component of adaptive social interactions, but little is known on its neural developmental correlates. We explored the role of amygdaloid complex (AC) and medial temporal lobe (MTL) in emotional memory recognition across development, by comparing fMRI activations of successful memory encoding of fearful and neutral faces in children (*n* = 12; 8–12 years) and adolescents (*n* = 12; 13–17 years). Memory for fearful faces was enhanced compared with neutral ones in adolescents, as opposed to children. In adolescents, activations associated with successful encoding of fearful faces were centered on baso-lateral AC nuclei, hippocampus, enthorhinal and parahippocampal cortices. In children, successful encoding of fearful faces relied on activations of centro-mesial AC nuclei, which was not accompanied by functional activation of MTL memory structures. Successful encoding of neutral faces depended on activations in anterior MTL region (hippocampal head and body) in adolescents, but more posterior ones (hippocampal tail and parahippocampal cortex) in children. In conclusion, two distinct functional specializations emerge from childhood to adolescence and result in the enhancement of memory for these particular stimuli: the specialization of baso-lateral AC nuclei, which is associated with the expertise in processing emotional facial expression, and which is intimately related to the specialization of MTL memory network. How the interplay between specialization of AC nuclei and of MTL memory structures is fundamental for the edification of social interactions remains to be elucidated.

## Introduction

Everyday social interactions imply efficient processing of facial emotional expressions but also accurate recognition memory of these stimuli. Enhanced memory for emotional faces is thus central to the development and maintenance of social skills. Recognition memory of emotional stimuli involves the activation of memory related structures in the medial temporal lobe (MTL), which are modulated by the activity of the baso-lateral nuclei of Amygdaloid Complex (AC), as first revealed in animals (Cahill and McGaugh, 1998; Dolcos et al., 2004; McGaugh, 2004; Sergerie et al., 2006; Roozendaal and McGaugh, 2011). While imaging studies have highlighted the structural development of MTL during childhood (Gogtay et al., 2006), few have studied its functional maturation in memory acquisition, and particularly the emotional modulation of recognition memory (Nelson et al., 2003; Vasa et al., 2011).

MTL structures, i.e., hippocampus and surrounding cortices, namely entorhinal, perirhinal, parahippocampal, and temporopolar cortices, are known to distinctly contribute to recognition memory in adults (Brown and Aggleton, 2001; Diana et al., 2007; Montaldi and Mayes, 2010; Wixted and Squire, 2011). According to recent models of declarative memory, perirhinal cortex receives afferent connections from the ventral stream and would be involved in item identification and familiarity-based recognition. On the other hand, parahippocampal cortex receives afferent connections from the dorsal stream and would be implied in the coding of object location and spatial context. Both perirhinal and parahippocampal cortices project to the entorhinal cortex, from which the fibers converge in the hippocampus. The hippocampus could thus be considered as a supra-structure, which binds item and context information, then leading to recollection of complex events (Brown and Aggleton, 2001; Diana et al., 2007). Interest for the development of the neural network of recognition memory is quite recent. fMRI studies have shown discrepant results about changes within MTL from childhood to adulthood. Some authors have reported decreasing activations in hippocampus and parahippocampal gyrus (Menon et al., 2005; Maril et al., 2010), whereas others found no age effect in hippocampus activation (Ofen et al., 2007; Maril et al., 2011). On the other hand, Ghetti et al. (2010) investigated the role of hippocampus and parahippocampal gyrus in detail recollection across ages (3 groups: 8 year olds, adolescents and adults). The groups were presented with black and white line drawings during a scanned encoding phase and later attempted to recall outside the scanner which color originally bordered the drawings. Correct recall of the surrounding color was considered as successful episodic detail recollection. Item recognition activated the hippocampus and posterior parahippocampal gyrus in 8 year olds, whereas these regions were specialized in detail recollection in adults. However, the structural maturation of the hippocampus is non-linear, encouraging to consider hippocampal subregions in developmental studies (Gogtay et al., 2006). Only one recent study has considered separately anterior and posterior hippocampal subregions in a comparison of 8–11 years old children and adults during source memory retrieval (Demaster and Ghetti, 2013). Data, acquired using the task of Ghetti et al. (2010), showed an age-related dissociation of hippocampal activity during successful episodic retrieval, with activity in the anterior hippocampus in adults but in the posterior one in children. Such developmental patterns of hippocampal function remain to be further explored to clarify the functional maturation of MTL network, and in particular the relationships between hippocampus, surrounding cortices and AC in the context of emotional modulation of memory.

The modulatory role of AC on the recognition memory network is well known in adults (Dolcos et al., 2004; Kensinger and Corkin, 2004; Sergerie et al., 2006; Murty et al., 2010). The memory modulation hypothesis proposes that amygdalar projections to the MTL declarative memory system are critical for consolidating memories of emotionally arousing events (McGaugh, 2004). This is favored by anatomical disposition of AC, anterior to and in continuity with the hippocampus, and by its numerous connections with cortical and sub-cortical areas engaged in memory. AC is a complex structure composed of several nuclei with distinct cytoarchitectony and connectivity: lateral nucleus, basal nucleus, central nucleus and cortico-mesial nucleus (Aggleton, 2000; LeDoux, 2007). Rodent studies have shown that (i) the basal nucleus is more specifically involved in fear conditioning (Sierra-Mercado et al., 2011); (ii) the lateral nucleus is more activated for learning associations between affect and stimuli (Johansen et al., 2011); (iii) the central nucleus would be at the crossroads of behavioral responses to painful stimulations (Kalin et al., 2004); and (iv) the mesial nucleus would be engaged in olfactory associations and sexual behavior (Lehman et al., 1980; Bian et al., 2008). The baso-lateral nuclei are involved in the modulation of memory-related brain activity in animals (Cahill and McGaugh, 1998; McGaugh, 2004; Roozendaal and McGaugh, 2011) and in memory recognition in human adults when using emotional pictures (Dolcos et al., 2004) and emotional facial expressions, mainly fear (Sergerie et al., 2006). These neuroimaging studies showed that the co-activation of the MTL and AC is critical to emotional memory formation. The localization of the modulated areas within MTL differs across studies, sometimes pointing to the hippocampal formation (Hamann et al., 1999; Dolcos et al., 2004; Kensinger and Corkin, 2004; Murty et al., 2009; St. Jacques et al., 2009) or to the entorhinal and perirhinal cortices in anterior MTL (Hamann et al., 1999; Dolcos et al., 2004; Ritchey et al., 2008) and parahippocampal cortex in posterior MTL (Kilpatrick and Cahill, 2003). Anterior hippocampus and surrounding cortices indeed have a high density of noradrenergic and glucocorticoid receptors which are thought to mediate AC's modulatory role on declarative memory (Roozendaal et al., 2009). Posterior MTL has been strongly implicated in contextual fear conditioning in both rodents (Rudy et al., 2004) and humans (Alvarez et al., 2008). Together, these findings support the memory modulation hypothesis. However, the development of neural networks linking recognition memory and emotional stimuli, especially the role of AC nuclei, during childhood, remains poorly understood.

The role of MTL in memory of emotional stimuli during adolescence has been underlined in two neuropsychological studies. The first one showed that, contrary to healthy adolescents, 11–15 years old patients with temporal lobe epilepsy displayed no emotional memory enhancement during learning of emotional word lists or recall of stories (Jambaqué et al., 2009). In the second study, Pinabiaux et al. (2013) compared memory recognition of emotional and neutral words and faces in a group of 8–18 years old patients with temporal lobe resection and a group of healthy age-matched participants. They found a deficit in emotional enhancement of memory in patients with temporal lobe resection for all emotional stimuli but fearful faces. Additionally, two fMRI studies have analyzed emotional modulation effects on the development of recognition memory networks in adolescents. Nelson et al. (2003) studied successful encoding of fearful, angry, happy and neutral faces in healthy 9–17 years old adolescents and adults. Memory enhancement for emotional faces was similar in adults and adolescents; surprisingly, in both groups, the amygdala was engaged bilaterally during successful encoding of neutral faces but not emotional faces (Nelson et al., 2003). According to the authors, methodological issues may explain why no activations of AC were found for fearful faces and why no age-related differences were observed. Indeed, there were four emotional expression conditions, and despite four presentations with different rating conditions (passive viewing, two emotional judgments, perceptual judgment) of the stimuli at encoding, the number of subsequent “hit” or “miss” trials for each emotional expression was reduced. More recently, Vasa et al. (2011) specifically analyzed, within hippocampus and AC, the activations associated with encoding of negative, positive and neutral pictures, using a procedure similar to that of Nelson et al. (2003). Activations were compared according to age (12–18 years old adolescents vs. adults) and memory accuracy (recalled vs. non-recalled pictures). Right AC was more engaged in adolescents than in adults for positive, but not for fearful, pictures (Vasa et al., 2011). No age-related changes were found in the hippocampus in either study, and no specific baso-lateral activations in AC were disclosed, in contrast to findings in adults (Dolcos et al., 2004; Sergerie et al., 2006).

In the present study, we sought to further characterize the age-related changes in the neural networks engaged during successful encoding of fearful and neutral faces in 8–12 years children and 13–17 years adolescents using a detailed analysis of Regions Of Interest (ROIs) in MTL structures (hippocampus head, body, and tail) extending to surrounding cortices (temporopolar, perirhinal, entorhinal, parahippocampal) and AC nuclei. We assumed that activations in AC and MTL memory related structures would vary with age when successfully encoding faces. In particular, we sought to investigate the involvement of baso-lateral AC nuclei in fear memory modulation from childhood. Considering the functional specialization of the hippocampus and parahippocampal gyrus previously reported during childhood, we expected to observe activations of more anterior regions of the MTL (hippocampal subregions and surrounding cortices) in adolescents compared with younger children. In addition we aimed to further decipher the functional organization within the MTL in relationship with amygdala nuclei.

## Materials and methods

### Participants

The study was approved by ethical committee (CPP Ile de France VI, Protocole INSERM C08-12, ID RCB: 2008-A00683, Paris, France) and informed consent was obtained from all parents and subjects.

A total of 24 healthy participants aged 12.87 ± 2.87 years (age range: 8–16.83 years, males/females: 13/11, 18 right handed) were included. None had history of neurological or psychiatric illness, and all participants completed the protocol. The population was split in two groups: children, from 8 to 12 years (*n* = 12, 5 girls, mean ± *SD* age = 10.46 years, ± 1.64 years) and adolescents, from 13 to 17 years (*n* = 12, 6 girls, mean age = 15.28 years ± 1.40 years).

### Stimuli

**Figure 1:**
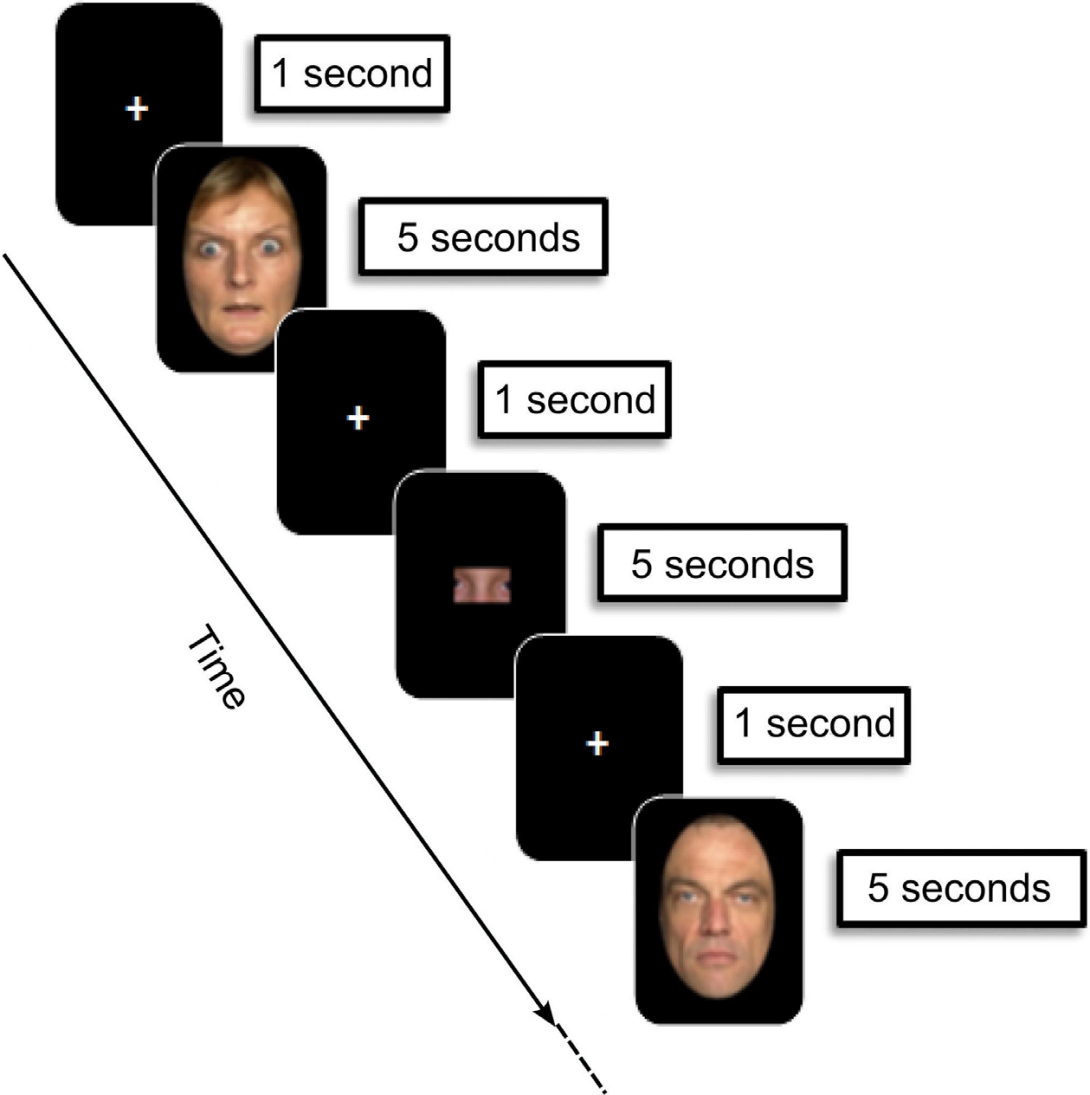
**Experimental design: encoding phase**. During each trial (12 s), children were asked to memorize faces presented (5 s), preceded and followed by a fixation cross (1000 ms). Then an image containing a “face part” was presented (5 s), and children were asked to indicate whether or not it belonged to the face presented immediately before. Faces were selected from the FACES database (Ebner et al., 2010).

Stimuli were 54 fearful and 54 neutral adult faces selected from the FACES database (Ebner et al., 2010). All faces belonged to different individuals in order to prevent subjects from subsequent false memory recognition of fearful/neutral stimuli sharing the same identity. To focus attention on facial emotional expression, we surrounded faces with a black mask, hiding hair, ears or neck as in previous studies (Golouboff et al., 2008; Pinabiaux et al., 2013) (Figure 1). Arousal was rated on a 5-point scale by an independent group of 15 young adults (mean age 22 years ± 1.78). As expected, fearful faces were rated as more arousing than neutral ones (mean rate: 1.52 ± 0.11 vs. 4.64 ± 0.9).

### Experimental design

The study was introduced to parents and children as a way to explore the neural basis of memory during development. The paradigm included two phases: (1) the encoding phase was performed in the MRI scanner using an event related fMRI paradigm followed by (2) the subsequent recognition phase performed outside the scanner. Participants were not informed of this subsequent recognition memory test. Before scanning, participants were trained in the encoding task on a laptop with different stimuli. For younger children, the training session also took place in a fake “Zero Tesla” MRI so they could become familiar with the machine, the noise, the response remote, and train to stay still.

Figure 1 displays the fMRI experimental design.

#### Encoding phase

The procedure was adapted from a task designed in a previous behavioral study of emotional memory in children and adolescents with temporal lobe epilepsy (Pinabiaux et al., 2013). The scanning session was divided into 6 runs of 24 trials each. Thirty-six fearful and 36 neutral faces were presented twice in a random order—run 1–3: first presentation; run 4–6: second presentation—to gain statistical power and to promote subsequent memory. An instruction screen was presented for 7200 ms at the beginning of each run. During each trial (12,000 ms), participants saw the face presented for 5000 ms, preceded and followed by a fixation cross for 1000 ms. Participants were asked to memorize the faces. To promote in depth encoding, the child was presented after each face with an image containing a “face part,” and was asked to indicate whether or not it belonged to the face presented immediately before by pressing a right (“yes”) or left (“no”) one-button remote (Figure 1). Images with parts of faces were constructed in such a way that they never contained a whole face attribute (eyes, nose, etc). A total of nine types of parts of each face were available. The part belonged to the previous face in half of the trials, and the different types of parts were balanced across trials. Experimental facial parts were chosen so that each type of part was balanced across the trials. The responses were qualitatively checked on line to control for attention, and trials without responses were discarded from analyses.

#### Recognition phase

An unexpected recognition memory test was performed outside the scanner 30 min after the end of the encoding phase. In this phase, the 72 previously presented faces and 36 foiled faces (18 fearful and 18 neutral) were randomly presented one by one on a computer screen. Faces were counterbalanced across subjects between being targets and foils. Participants judged each item as new or old using the keyboard, without time constraints.

### Imaging procedure

Images were acquired on a 3-T Siemens Magnetom Trio scanner. High resolution 3D IR–prepped T1-weighted anatomic scans were first acquired (repetition time = 2300 ms, echo time = 3 ms, TI : 900 ms, 256-mm field of view, matrix 240 × 256, 160 sagittal slices, 1 × 1 × 1.1 mm3) in 7 min 46 s. During encoding, 123 T2^*^-weighted EPI images were acquired per run (TR = 2400 ms, TE = 30 ms, 192-mm FOV, 64 × 64 matrix, 81 flip angle, 40 axial slices, 3 mm isotropic, 5 min 2 s). The three initial volumes were discarded to allow for T1 equilibration.

Data were analyzed using SPM5 (http://www.fil.ion.ucl.ac.uk/spm/). Differences in slice acquisition timing were corrected by resampling all slices in time to match the middle slice. Functional volumes were spatially realigned to correct for motion artifacts. Scans with more than 4 mm movement in one direction were discarded. Images were then spatially normalized using a pediatric template based on matched reference data of NIH MRI study of normal brain development created with the TOM toolbox (Wilke et al., 2008). This template was automatically generated in a SPM5 toolbox using an algorithm including age and gender of each of our participants. Finally, images were smoothed using a 5-mm isotropic Gaussian filter.

### Data analysis

#### Behavioral data

Data were analyzed with Statistica (www.statsoft.com). Percents of correct and false recognitions were collected and averaged in each age group and a corrected memory accuracy measure was computed as %hits-%false recognition. Global effects of age (8–12 years vs. 13–17 years) were analyzed using 2 by 2 Mann–Whitney tests. Global effects of emotional expression (fearful vs. neutral) were analyzed within each group separately using 2 by 2 Wilcoxon tests. Group x emotional expression interaction was also explored using 2 by 2 Wilcoxon tests. Accuracy on the “face parts” task and interaction between encoding and recognition accuracy were also examined using non-parametric comparison and correlation.

#### fMRI data

Individual GLM–based analyses were conducted with SPM5 (http://www.fil.ion.ucl.ac.uk/spm/). Trials were categorized in four types according to both emotional expression and recognition status (fearful hits, fearful misses, neutral hits, and neutral misses) based on individual memory performance and regressor functions were constructed for each trial type.

***Whole brain analysis***. Individual contrasts of interest for fearful and neutral faces (Dmfear and Dmneutral) were based on the differential neural activity on a common memory contrast defined by Dm = hits-misses (Paller and Wagner, 2002), using a fixed-effects model across the six sessions. Second-level group analyses used a mixed-effects (MFX) model implemented in the DISTANCE toolbox of SPM5 (Mériaux et al., 2006; Roche et al., 2007). This MFX model takes into account the error measure on blood oxygenation level–dependent (BOLD) contrast in data with high inter-subject variability (Mériaux et al., 2006; Roche et al., 2007). Permutation *t*-tests (one million of permutations) were computed to compare Dmfear and Dmneutral between 8 and 12 years and 13 and 17 years groups. Permutation analyses were conducted under a non-parametric assumption and corrected for multiple comparisons (Holmes et al., 1996). Whole brain Dmfear and Dmneutral contrast maps were compared between groups using, at the voxel level, an uncorrected height threshold of *p* < 0.005, and a cluster size threshold of >5 contiguous voxels (de Vanssay-Maigne et al., 2011).

Eight ROIs were manually delineated in the MTL on both sides of each subject, as described in previous studies (Noulhiane et al., 2006, 2007; de Vanssay-Maigne et al., 2011): AC, hippocampus head, body, and tail, entorhinal, perirhinal, parahippocampal and temporopolar cortices. The ROI boundaries were identified using anatomical landmarks (Insausti et al., 1998; Duvernoy and Bourgouin, 1999; Pruessner et al., 2002) taking into account hippocampus development (Insausti et al., 2010). The protocol consisted of a volumetric analysis based on histological landmarks reported on T1-MRI, to offer pertinent MRI landmarks. Because AC nuclei are not visible on T1-MRI, we adopted a methodology previously used by Dolcos et al. (2004) consisting in dividing AC in four quadrants. To delineate the ROIs, the protocol implied to progress in a rostro-caudal direction along the MTL in a coronal plane (1 mm section) while checking the delimitation in the other planes (axial, sagittal, 3D). To account for age-dependent volume changes, ROIs were separately manually drawn on three subjects of each group (the youngest, the oldest and the median). ROIs from these subjects were then normalized to create a template. Mean contrast values of fear hits, fear misses, neutral hits and neutral misses were extracted from ROIs in each subject using MarsBar toolbox (http://marsbar/sourceforge.net). For each subject, BOLD response was contrasted between subsequent recognized faces (Hits) and fixation trials, and between subsequent non-recognized faces (Misses) and fixation trials, within each ROI. First, global effects of subsequent memory (Hits > Misses) were analyzed within each group for fearful and neutral faces separately using 2 by 2 Wilcoxon tests. Group (8–12 years vs. 13–17 years) × subsequent memory (Hits vs. Misses) interaction was then explored within regions showing subsequent memory effects, using 2 by 2 Mann–Whitney tests. Due to the multiple ROIs approach (MTL: *n* = 16; AC nuclei: *n* = 4), thresholds were corrected for multiple comparisons (Bonferroni's adjustement). *P*-values have been adjusted in accordance with the number of ROIs separately for MTL and AC analyses. Thus adjusted *p*-values correspond to p/16 for MTL (8 ROIs × 2 hemispheres) and p/4 for AC (2 ROIs × 2 hemispheres), and were compared to α = 0.05 threshold.

## Results

### Behavioral results

**Figure 2:**
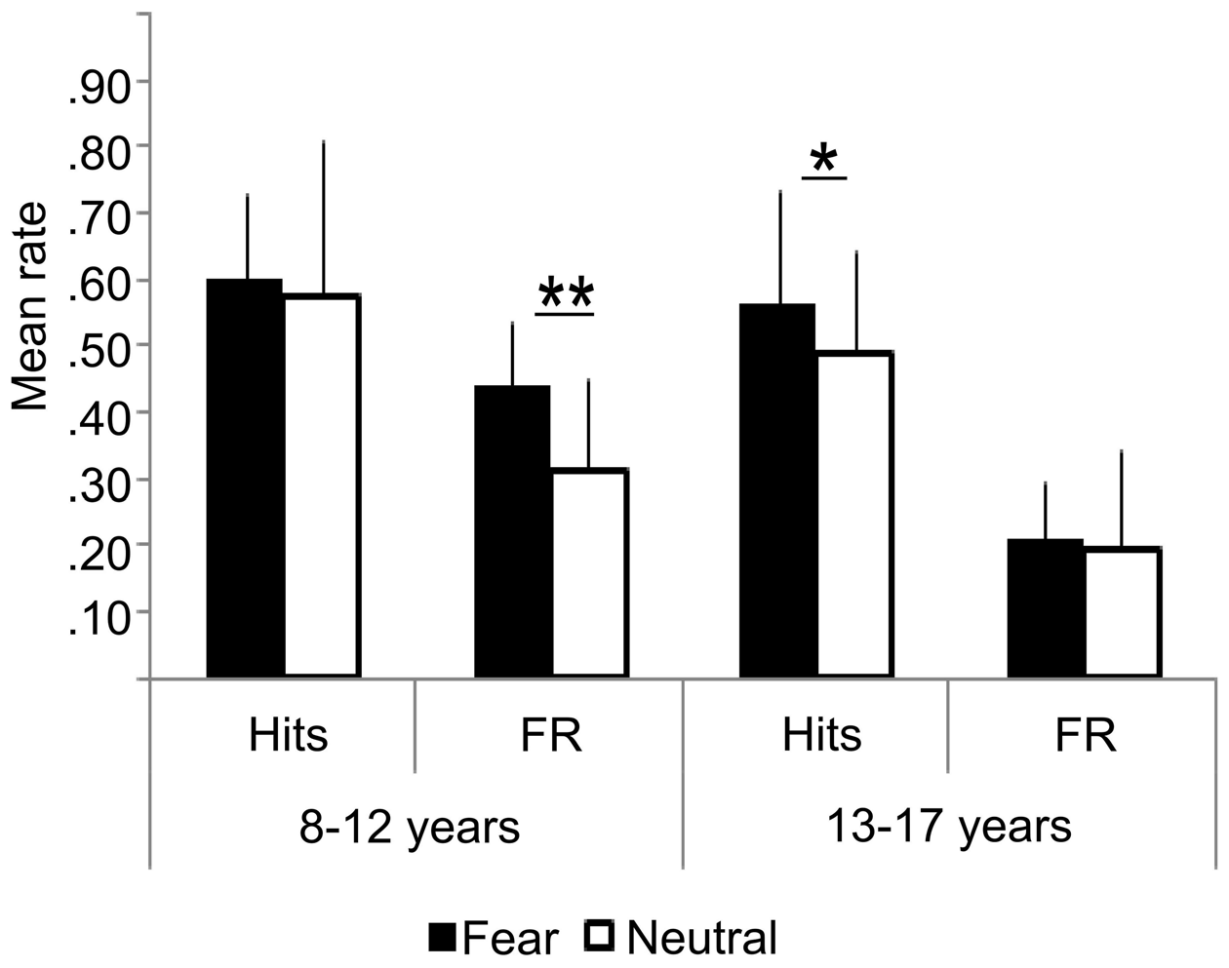
**Behavioral results: hits, False Recognition (FR) for fearful and neutral faces in children (8–12 years) and adolescents (13–17 years)**. Error bars = mean square errors. Significant differences between fearful and neutral conditions are indicated as follows: ^*^p < 0.05; ^**^p < 0.01. Fearful faces were more accurately recognized than neutral faces in the adolescents group, but not in the children group. There were more false recognitions of fearful faces than neutral ones in the children group only.

Figure 2 presents the behavioral performances on the recognition memory task of the two age groups. All participants performed well above chance level at the ‘face parts’ task during encoding (accuracy range: min = 72%–max = 89%). There were no age-related differences, neither for “face parts” task for fearful (Mean_13−17_ = 0.82 ± 0.08; Mean_8−12_ = 0.79 ± 0.05; *Z* = 1.17, ns), nor for neutral faces (Mean_13−17_ = 0.85 ± 0.08; Mean_8−12_ = 0.80 ± 06; *Z* = 1.6, ns). We thus assume that children and adolescents paid attention to the faces equivalently during the scanning session. There was no significant relationship in accuracy between encoding and recognition tasks, whether for fearful or neutral faces (rho = −0.088; ns; rho = −0.12; ns, respectively).

Percent of hits was similar in both groups (fearful: *Z* = 0.72, ns; neutral: *Z* = −1.22, ns), but age and emotional expression significantly interacted during the recognition test. Fearful faces were more accurately recognized than neutral faces in adolescents (Mean_Fear_ = 0.56 ± 0.13 vs. Mean_Neutral_ = 0.49 ±0.24, *Z* = 2.25, *p* = 0.024), but not in children (Mean_Fear_ = 0.59 ± 0.10 vs. Mean_Neutral_ = 0.58 ± 0.14; *Z* = 0.67, ns). In children, there was more false recognition of fearful faces than neutral ones (0.41 vs. 0.29, *Z* = 2.63, *p* = 0.0086), whereas equivalent recognition was observed in adolescents (0.23 vs. 0.20, *Z* = 0.66, ns). Consequently, an interaction between groups and emotional expressions was observed on corrected memory accuracy measure: it was better for fearful than neutral faces in the adolescent group (Mean_Fear_ = 0.39 ± 0.13 vs. Mean_Neutral_ = 0.30 ± 0.14, *Z* = 2.58, *p* = 0.0099), whereas no difference was observed in the children group (Mean_*Fear*_ = 0.19 ± 0.17; Mean_Neutral_ = 0.30 ± 0.10, *Z* = 1.27, ns).

### Neuroimaging results

#### Whole brain analysis

Table 1 shows the brain regions where activations corresponding to DmFear were sensitive to age-related changes (respectively 13–17 years > 8–12 years and 8–12 years > 13–17 years). In 13–17 years, regions specifically sensitive to subsequent memory for fearful faces were right hippocampal body and tail, left AC, more specifically baso-lateral nuclei (x: -24; y: 0; z: −24), and right thalamus (x: 18; y: −36; z: 12). Conversely, a larger network was specifically activated in 8–12 years group compared to 13–17 years group, comprising bilateral frontal gyri, right middle temporal gyrus, bilateral superior parietal lobule, cingulum, left caudate, bilateral cuneus, precuneus, fusiform and middle occipital gyri, and right cerebellum.

**Table 1:** **Neural substrates of subsequent memory effect (Dm) for fearful faces across ages**.

**Regions**	***X***	***Y***	***Z***	**Cluster size**	***Z*-value**	***p***
**13–17 YEARS > 8–12 YEARS**						
Left amygdaloid complex (Baso-lateral nuclei)	−24	0	−24	6	2.967	0.00151
Right hippocampal body	33	−27	−6	6	3.031	0.00122
Right hippocampal tail	39	−33	−6	5	2.705	0.00342
Right thalamus	18	−36	12	11	2.863	0.00210
**8–12 YEARS > 13–17 YEARS**						
Right amygdaloid complex (Centro-mesial nuclei)	21	0	−15	10	3.487	0.00024
Left amygdaloid complex (Centro-mesial nuclei)	−18	−3	−18	11	3.487	0.00024
Right inferior frontal gyrus	45	21	3	5	3.487	0.00024
Left inferior frontal gyrus	−42	45	−12	5	3.487	0.00024
Right middle frontal gyrus	24	21	51	13	3.297	0.00049
Left middle frontal gyrus	−3	54	−9	13	3.487	0.00024
Right superior frontal gyrus	18	48	3	30	3.297	0.00049
Right middle temporal gyrus	63	−33	−15	33	3.487	0.00024
Right superior parietal lobule	30	−75	48	11	3.297	0.00049
Right fusiform gyrus	30	−48	−18	5	3.297	0.00049
Left fusiform gyrus	−36	−57	−18	5	3.182	0.00073
Right precuneus	6	−48	9	7	3.297	0.00049
Left precuneus	−15	−81	45	9	3.487	0.00024
Left cuneus	−3	−99	12	11	3.487	0.00024
Right lingual gyrus	24	−66	45	6	2.815	0.00244
Left lingual gyrus	−6	−72	−9	6	3.297	0.00049
Left inferior occipital gyrus	−30	−93	−12	43	3.487	0.00024
Right middle occipital gyrus	33	−81	12	90	3.487	0.00024
Left middle occipital gyrus	−45	−72	9	50	3.487	0.00024
Anterior cingulum	−3	27	30	6	3.031	0.00122
Middle cingulum	3	−3	36	8	3.182	0.00073
Posterior cingulum	3	36	27	18	3.297	0.00049
Right cerebellum	3	−72	−30	42	3.487	0.00024

Whole brain analysis. Non-parametric group comparison (13–17 years > 8–12 years and 8–12 years >13–17 years) of Dm (Dm = Hits-Misses) for fearful faces (p < 0.005; 5 contiguous voxels).

Table 2 shows the regions where activations corresponding to DmNeutral were sensitive to age-related changes (respectively 13–17 years > 8–12 years and 8–12 years > 13–17 years). These comparisons showed stronger activations in 13–17 years in bilateral inferior temporal gyrus and right precuneus. Activations in right middle frontal, middle occipital and fusiform gyri, and left cuneus were more important in 8–12 years.

**Table 2:** **Neural substrates of subsequent memory effect (Dm) for neutral faces across ages Whole brain analysis**.

**Regions**	***X***	***Y***	***Z***	**Cluster size**	***Z*-value**	***p***
**13–17 YEARS > 8–12 YEARS**						
Right inferior temporal gyrus	54	−63	−15	8	3.487	0.00024
Left inferior temporal gyrus	−48	−54	−15	6	3.487	0.00024
Right precuneus	33	−69	36	5	3.487	0.00024
**8–12 YEARS > 13–17 YEARS**						
Right middle frontal gyrus	27	3	51	10	3.297	0.00049
Right fusiform gyrus	36	−57	−12	6	3.297	0.00049
Left cuneus	−18	−93	3	5	3.297	0.00049
Right middle occipital gyrus	36	−84	9	8	3.031	0.00122

Non-parametric group comparison (13–17 years > 8–12 years and 8–12 years > 13–17 years) of Dm (Dm = Hits-Misses) for neutral faces (p < 0.005; 5 contiguous voxels).

**Figure 3:**
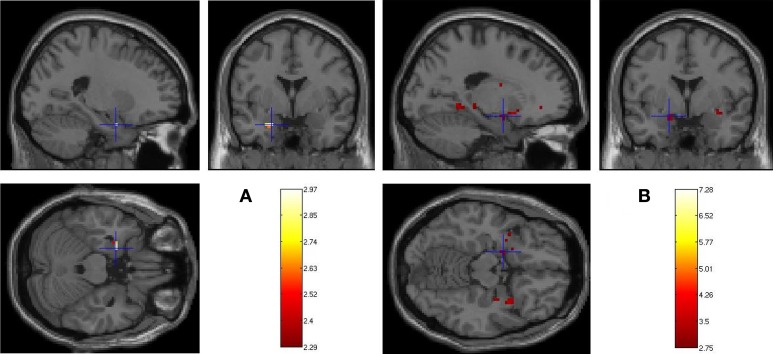
**Age-related changes in fearful faces successful encoding**. Whole brain activations associated with Dm (Dm = Hits-Misses) centered on amygdaloid complex for **(A)** 13–17 > 8–12 years (left basolateral nuclei: −24, 0, −24) and **(B)** 8–12 > 13–17 years (left centromesial nuclei: −18, −3, −18) contrasts (*p* < 0.005, 5 contiguous voxels).

To sum up, (i) DmFear contrast revealed that MTL regions were specifically sensitive to subsequent memory for fearful faces in 13–17 years groups, but not in the 8–12 years groups in which the network was larger (activations centered on amygdaloid nuclei are presented in Figure 3); (ii) DmNeutral contrast showed no MTL nor AC activations in either group.

#### MTL ROI analysis

**Figure 4:**
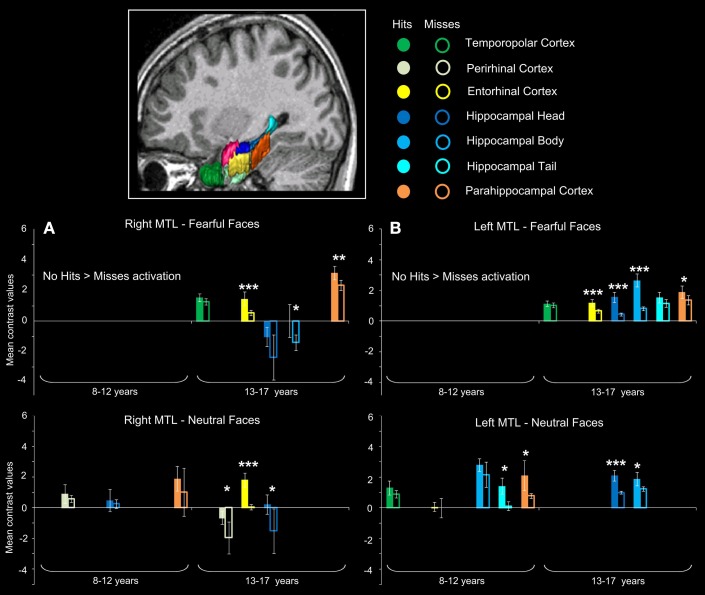
**Subsequent memory activations (Hits > Misses) in medial temporal lobe structures during emotional face encoding**. Mean contrast values (Hits and Misses) by age group for fearful and neutral faces within ROIs in right **(A)** and left **(B)** medial temporal lobe (MTL). Bars represent mean square errors. Significant levels are indicated as follows: ^*^p < 0.01; ^**^p < 0.001; ^***^p < 0.0005. In 8–12 years group, left hippocampal tail (*p* < 0.00016), left parahippocampal cortex (*p* = 0.00248) were sensitive to subsequent memory for neutral faces, whereas no region was sensitive to subsequent memory for fearful faces. In 13–17 years group, fearful faces memory effects were seen in bilateral entorhinal cortices (*p*s < 0.00016), left hippocamal head (*p* < 0.00016), bilateral hippocampal bodies (right: *p* = 0.0077; left: *p* < 0.00016) and bilateral parahippocampal cortices (right: *p* = 0.00064; left: *p* = 0.0048); neutral memory effects were found in right temporopolar cortex, bilateral entorhinal cortices (*p*s < 0.00016), right perirhinal cortex (*p* = 0.0054), bilateral hippocampal heads (right: *p* = 0.0088; left: *p* < 0.00016) and left hippocampal body (*p* = 0.0015).

Figure 4 presents the regions showing memory effect (i.e., Hits > Misses) (see Figure S1 in Supplementary Material for the report of Misses > Hits activations). We found no significant effect of subsequent memory (i.e., Hits > Misses) of fearful faces in children.

For neutral faces, some regions were sensitive to subsequent memory [left hippocampal tail (*p* < 0.00016), left parahippocampal cortex (*p* = 0.00248)]. By contrast, several regions were sensitive to subsequent memory of both fearful and neutral faces in the 13–17 years group. Fearful faces memory effects were seen in bilateral entorhinal cortices (*p*s < 0.00016), left hippocampal head (*p* < 0.00016), bilateral hippocampal bodies (right: *p* = 0.0077; left: *p* < 0.00016) and bilateral parahippocampal cortices (right: *p* = 0.00064; left: *p* = 0.0048). Neutral faces memory effects were found in right temporopolar cortex, bilateral entorhinal cortices (*p*s < 0.00016), right perirhinal cortex (*p* = 0.0054), bilateral hippocampal heads (right: *p* = 0.0088; left: *p* < 0.00016) and left hippocampal body (*p* = 0.0015). Age group (8–12 years vs. 13–17 years) × memory (Hits vs. Misses) interaction analysis was conducted separately for fearful and neutral faces and confirmed that memory effects (Hits > Misses) were greater for fearful faces in 13–17 years group than in 8–12 years group within bilateral entorhinal cortices, left hippocampal head, bilateral hippocampal bodies and bilateral parahippocampal cortices (*p*s < 0.01), and for neutral faces within right temporopolar cortex, bilateral entorhinal cortices, right perirhinal cortex, bilateral hippocampal heads and left hippocampal body (*p*s < 0.01).

#### Amygdaloid complex ROI analysis

**Figure 5:**
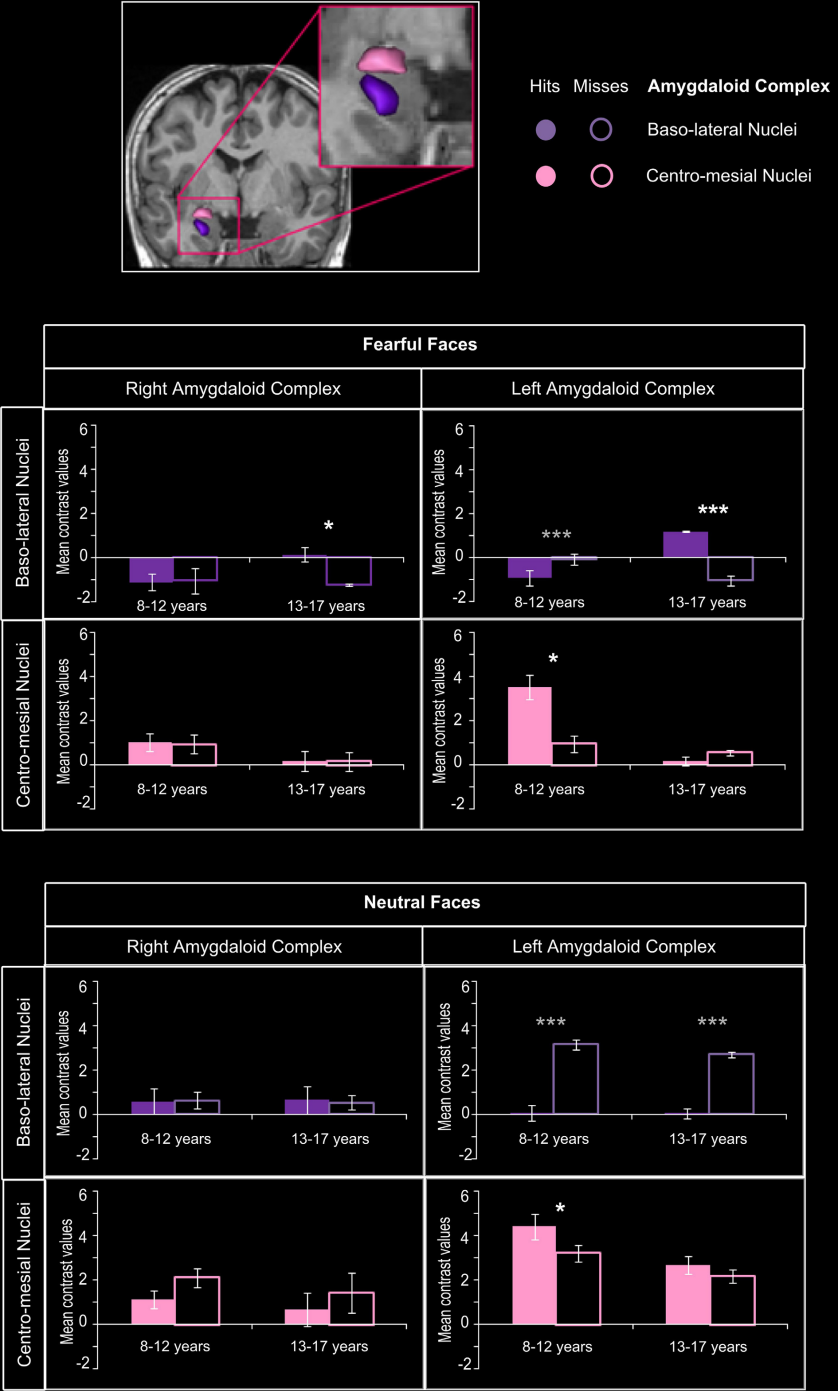
**Amygdaloid complex activations during emotional face encoding**. Mean contrast values (Hits and Misses) by age group for fearful and neutral faces within baso-lateral and centro-mesial nuclei of right and left amygdaloid complex (AC). Bars represent mean square errors. Significant levels are indicated in white for subsequent memory effect (Hits > Misses) or in gray (Misses > Hits) as follows: ^*^p < 0.01, ^***^p < 0.0005. In the 8–12 years group, a subsequent memory effect was found in centro-mesial nuclei of left AC for fearful (*p* = 0.0027) and neutral faces (*p* = 0.0034), but no such effect was seen in baso-lateral nuclei. Inversely, in the 13–17 years group, baso-lateral nuclei on both sides were specifically sensitive to fearful subsequent memory effects (right: *p* = 0.0034; left: *p* < 0.00004), whereas no significant effect was found for neutral faces in AC nuclei.

Figure 5 presents the mean contrast values with hits and misses in amygdaloïd complex. In the 8–12 years group, a significant memory effect (Hits > Misses) was found in centro-mesial nuclei of left AC for fearful (*p* = 0.0027) and neutral faces (*p* = 0.0034), but no such effect was seen in baso-lateral nuclei. Inversely, in the 13–17 years group, baso-lateral nuclei on both sides were specifically sensitive to fearful memory effects (right: *p* = 0.0034; left: *p* < 0.00004), whereas no significant Hits > Misses effect was found for neutral faces in AC nuclei.

For fearful faces, age group (8–12 vs. 13–17 years) × memory (Hits vs. Misses) interaction analysis revealed that subsequent memory effects were greater in 13–17 years group than in 8–12 years group within right and left baso-lateral nuclei (*p*s < 0.00004), but greater in 8–12 years group than in 13–17 years group within left centro-mesial nuclei (*p* < 0.00004). A significant interaction was found for neutral faces within left centro-mesial nuclei, subsequent memory effects of neutral faces being more important in 8–12 years than in 13–17 years (*p* < 0.0024).

## Discussion

The aim of this study was to clarify age-related changes in neural networks involved in encoding memory of fearful faces, and especially the role of the AC in modulating memories across development. Behavioral data showed that, when compared with memory of neutral faces, memory for fearful faces was enhanced in adolescents but not in children. This difference was associated with two functional developmental specializations: (i) a specialization of baso-lateral AC nuclei for specific encoding of fearful faces in adolescents, but a non-specific involvement of centro-mesial AC nuclei in children; (ii) the specialization of distinct parts of the hippocampus and surrounding cortices in recognition memory processes, depending on the emotional content: whereas MTL activation was associated with encoding of fearful faces in adolescents only, a rostro-caudal segregation was observed for neutral stimuli in children, with more posterior activations. Finally, with age, extra-MTL regions were less engaged, reflecting an ‘economic’ dynamic of networks across development, i.e., relying on a smaller number of ultra-specialized structures. Importantly, these functional activity changes reflect the natural plasticity of neural memory networks during development rather than a modification in memory accuracy or in attentional engagement during the encoding task between age groups.

### Emotional specialization of AC nuclei with age

We demonstrate a developmental switch in the involvement of AC nuclei during successful encoding of faces. Successful encoding of fearful faces specifically relied on activity of left baso-lateral AC nuclei in adolescents, who thus presented an adult-like pattern of emotional memory related amygdala activity (Dolcos et al., 2004; Sergerie et al., 2006). In animals and human adults, baso-lateral AC nuclei are especially implicated in emotional modulation of memory encoding in hippocampus (LaBar and Cabeza, 2006; Roozendaal and McGaugh, 2011). By contrast, we show that children engaged centro-mesial AC nuclei during successful encoding of both fearful and neutral faces. In light of their anatomical arrangement, cytoarchitecture and connectivity with the hypothalamus, the involvement of centro-mesial AC nuclei in children may reflect the engagement of low level processes (Lehman et al., 1980; Kalin et al., 2004; Bian et al., 2008). Indeed, while our data suggest that the involvement of baso-lateral AC nuclei in adolescents translates into a higher level of expertise in processing emotional facial expression, the activation of centro-mesial AC nuclei would be correlated with the immaturity of such social skill (Guyer et al., 2008). That, in 8–12 years children, centro-mesial AC nuclei activation was not specific of fearful faces may explain why this age group showed no emotional enhancement of memory. Accordingly, stronger activation of AC has been associated with neutral faces in 11 year-old children, whereas, in adults, AC was more activated when viewing fearful faces (Thomas et al., 2001). More recently, AC was found to be more activated in adolescents than in adults during passive and active processing of emotional facial expressions (Passarotti et al., 2009), notably fear (Guyer et al., 2008). However, owing to the anatomical complexity of AC substructures and of their functional segregation, it is surprising that no study so far has addressed the role of the activation of AC nuclei. We show here a functional developmental specialization of AC nuclei related to fearful faces memory. On the one hand, this study brings new evidence that emotional enhancement of fearful faces memory relies on the specialization of baso-lateral nuclei of AC in emotional memory modulation, which appears during adolescence. This may be particularly true for fearful faces, since AC is especially sensitive to this type of emotional stimuli (McGaugh, 2004). Evidence of baso-lateral AC nuclei activations for other emotional stimuli during adolescence is however needed. On the other hand, the proposition of non-specific activity of centro-mesial AC nuclei in children should be explored in further functional and/or anatomical studies focusing on the distinct role of AC nuclei in emotional faces processing across development.

### Memory specialization of MTL with age

We bring new evidence about the role of MTL structures in encoding fearful and neutral faces during development, thanks to a specific MTL ROIs approach combined with a sensitive paradigm using facial expressions. Previous developmental fMRI studies of emotional memories did not elicit age-related differential hippocampal activity (Nelson et al., 2003; Vasa et al., 2011), while those of recognition memory showed an age-related decrease of hippocampal activity (Menon et al., 2005; Maril et al., 2010). Based on anatomical evidence (Gogtay et al., 2006) and on recent functional findings (Demaster and Ghetti, 2013), we looked at age-related differences in hippocampal head, body and tail, using a dedicated ROI analysis. Using an fMRI source-memory task, Demaster and Ghetti (2013) have demonstrated a shift of activity during episodic retrieval, from the posterior hippocampus in 8–11 years old children toward the anterior hippocampus in adults. We further demonstrate that age-related contributions of hippocampal sub-regions depend on emotional expression. The successful encoding of neutral faces was associated with a stronger activity in the posterior part of the MTL (hippocampal tail and parahippocampal cortex) in children, whereas it was related to greater activations in the anterior one (hippocampal head and body) in adolescents. This developmental change in functional engagement along a longitudinal axis in MTL is thus congruent with DeMaster and Ghetti's results (2013), although stimuli are intrinsically different (faces vs. drawings) and the tasks did not request the same cognitive resources (encoding vs. source memory). Individual longitudinal studies may greatly help in the description of this developmental shift. When dealing with fearful faces, age-related MTL activation changes were different with a “all or nothing” pattern of activations, rather than a rostro-caudal age-related segregation. Indeed, no effect of subsequent memory for fearful faces was found within MTL in children, whereas bilateral hippocampus and parahippocampal gyrus (entorhinal and parahippocampal cortices) were activated in adolescents. A lack of interplay between centro-mesial AC nuclei and MTL in children would be congruent with the absence of emotional memory enhancement in behavioral results, and is also illustrated by the non-specific centro-mesial activation of AC during successful encoding of both fearful and neutral faces in children. Eventually, adolescents thus display a more “adult-like” pattern of behavior and activations, involving baso-lateral AC, hippocampus and parahippocampal gyrus (Dolcos et al., 2004; Sergerie et al., 2006).

### Relationship between specializations of AC nuclei and MTL across development

**Figure 6:**
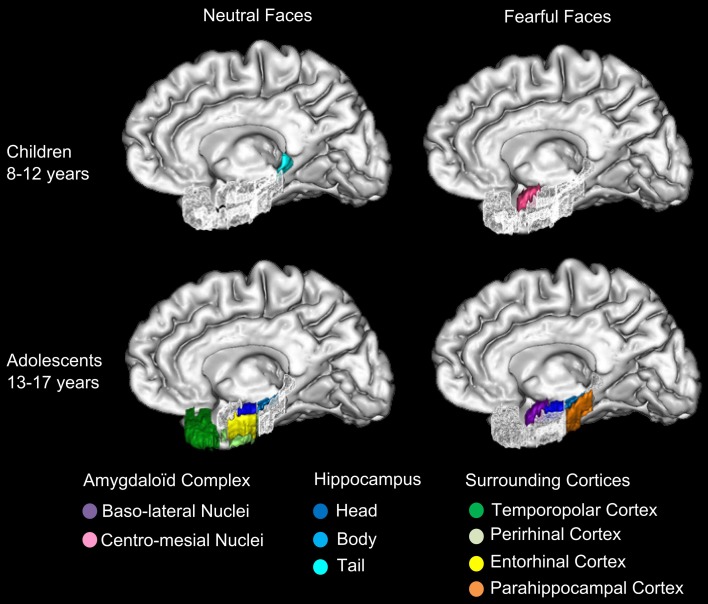
**Specializations of amygdaloid complex nuclei and MTL for neutral and fearful faces across groups**. The specialized activations of baso-lateral amygdaloid nuclei (AC) and medial temporal lobe (MTL) memory network are schematized in this figure. In 8–12 years children, centro-mesial amygdaloid complex nuclei are activated for successful encoding of both neutral and fearful faces and hippocampal tail is activated for successful encoding of neutral faces. In 13–17 years adolescents, successful encoding of fearful faces is associated with activations of baso-lateral amygdaloid complex nuclei, hippocampal head and tail and parahippocampal gyrus, whereas encoding of neutral faces is associated with activations of hippocampal head and body, temporopolar, perirhinal and entorhinal cortices. The developmental shift from the engagement of centro-mesial AC nuclei in children to baso-lateral AC nuclei in adolescents may reflect the growing expertise in processing emotional facial expression, which may enhance the memory for such stimuli.

This study shows that the specialized activations of baso-lateral AC nuclei and MTL memory network are associated with the emergence of emotional memory modulation in adolescents. Indeed, the developmental shift from the engagement of centro-mesial AC nuclei in children to baso-lateral AC nuclei in adolescents could reflect the growing expertise in processing emotional facial expression, which may enhance the memory for such stimuli (Figure 6). At least two alternative interpretations can be suggested. The first is a “cascade explanation,” in which the specialization of baso-lateral AC nuclei constitutes a substantial gateway to MTL memory network, resulting in the emergence of emotional memory modulation. Data from Guyer et al. (2008) support this assumption, as these authors observed a co-activation of AC and hippocampus in association with perception of fearful faces in 9–17 year-old adolescents. The second is an “interplay explanation,” in which the specializations of baso-lateral AC nuclei and MTL memory network emerge concomitantly. Neuropsychological studies in adults with temporal lobe epilepsy (Glogau et al., 2004; Carvajal et al., 2009) and in adolescents after temporal lobe resection (Pinabiaux et al., 2013) have shown that perception and encoding of emotional faces are correlated. Indeed, patients with temporal lobe epilepsy show impairments in perception of facial expressions, which are more important in early onset epilepsy (Meletti et al., 2003; Golouboff et al., 2008; Sedda et al., 2013). In the “interplay explanation,” enhanced memory for emotional faces would be a determinant of growing expertise in facial processing. Accordingly, accurate memory for a fearful face would later result in more accurate processing of this face. In the case of memory for fearful faces, this agrees with the appraisal theory of emotion (Brosch et al., 2010) which suggests that fear is a more relevant emotion than happiness for species survival and social behavior. Future studies using other emotional facial expressions are needed to confirm this assumption about the age-related enrolment of AC nuclei in emotional modulation of memory. Especially, functional connectivity analyses would help to better understand how the interplay between AC and MTL specializations establishes across development.

### Extra-MTL activations

Whole brain analyses (Supplementary material) showed that extra-MTL areas were more activated in children than in adolescents. Parieto-occipital cortices were more engaged in children during successful encoding of fearful faces, possibly reflecting the involvement of perceptual systems (Maril et al., 2011). Ventral stream, cuneus and precuneus activations indicate that successful encoding of fear faces may require more visual attentional resources and visuo-spatial imagery in children compared with adolescents (Cavanna and Trimble, 2006; Murty et al., 2010). Frontal areas also were more activated in children when encoding fearful faces. Again, this may stand for the higher attentional engagement in younger children, given our encoding task. This network may subtend memory along with the centro-mesial AC nuclei until the MTL becomes mature enough to sustain efficient long-term memory. By contrast, the only regions more activated in adolescents were in the MTL. It thus seems that adolescence is truly characterized by a switch toward a network centered on baso-lateral AC and its interactions with MTL memory system, like in adults. Alternatively, this greater involvement of the emotional system involving AC may agree with neurobehavioral models describing the imbalance between cognitive/regulatory systems involving frontal areas and sub-cortical emotional systems involving AC as a characteristic of adolescence (Nelson et al., 2005; Ernst et al., 2006; Casey et al., 2008; Guyer et al., 2008; Passarotti et al., 2009). Indeed, prefrontal areas are involved in memory of emotional faces in adults (Sergerie et al., 2005). That we found no specific frontal activation in adolescents contrary to children may thus be a peculiarity of this period of development; if and how frontal involvement evolves between adolescence and adulthood remains, however, unclear.

### Methodology issues and study limitations

First, we had to deal with some variability of BOLD signal in children. The use of non-parametric analyses allowed us to compute group comparisons for small samples without normality assumption (Mériaux et al., 2006; Roche et al., 2007). Our definition of age group was stated *a priori*, in order to constitute homogeneous groups in terms of number of subjects (*n* = 12) and age range (4 years), but also because behavioral studies have indicated that rapid improvements in episodic memory occur during middle and late childhood (Brainerd et al., 2004; Ghetti and Angelini, 2008). This age-group split is congruent with neuroimaging results obtained by Ghetti et al. (2010) showing that there was a qualitative change in memory related activations within MTL between 11 year-old children and 14 year-old adolescents, who presented a more adult-like pattern of activations. With a larger number of subjects, however, age could have been considered as a continuous variable and regression analyses could have been conducted without any *a priori* statement on age-groups definition. Indeed, our results point to age-related changes around 12–13 years, but more gradual changes may occur within 9–17 years range. Studies exploring longitudinal intra-individual changes are needed to demonstrate such progressive changes in the functional bases of memory, but are exceedingly difficult to conduct.

Second, in a preliminary pilot study, we had used children and adolescents faces (5–15 years old) from our own database (Golouboff et al., 2008; Pinabiaux et al., 2013), but we had been unable to show subsequent memory effects due to an insufficient number of item per conditions (fear hits, fear misses, neutral hits, neutral misses). We thus employed adult faces (young, middle age and older) from a larger database (Ebner et al., 2010) ignoring the reported own-age bias, in which people recognize faces of people of their own age more accurately than faces of other ages (e.g., Anastasi and Rhodes, 2005). Nevertheless, recent studies have shown that this age effect changes rapidly with age (Hills and Lewis, 2011) and that the face recognition system is updated on the basis of recent experience and/or motivation to process faces (Hills, 2012). We can assume that the use of adults' faces puts our two experimental groups on an equal level regarding the own-age bias, which might not have been true with a 5–15 years range database.

Finally, a recent study pointed out that the use of adult-sized head coils on child-sized heads may lead to underestimation of the signal-noise ratio, especially in mesial brain regions (McKone et al., 2012). Thus, one may say that age-related activations within MTL may be explained by bigger distance (i.e., smaller heads) between brain and coils in children with respect to adolescents. However, brain growth is largely achieved by the age 7 and very marginal after age 11, making it unlikely to create significant differences between groups (Giedd et al., 2006).

## Conclusion

Memory for fearful faces is enhanced when compared with that for neutral ones in adolescents, but not in children. During adolescence, neural networks get similar to that of adults, involving MTL key structures, namely baso-lateral AC nuclei, hippocampus and parahippocampal gyrus. In children, however, encoding of fearful faces relies on large frontal and posterior activations and on the engagement of centro-mesial AC nuclei, that is not accompanied by activation of memory structures in MTL or by behavioral enhancement of memory. The specific activation of baso-lateral AC nuclei during adolescence is thought to accompany a higher level of expertise in processing emotional stimuli. Whether memory bias for fearful faces results *from or in* expertise in the processing of fearful expressions remains to be explored. Future effective functional connectivity analysis would be helpful to investigate this issue, using stimuli and procedure which have been validated with the present study.

## Authors contributions

Charlotte Pinabiaux, Isabelle Jambaqué, Lucie Hertz-Pannier, Sébastian Rodrigo, and Catherine Chiron conceived the experiments. Charlotte Pinabiaux and Marion Noulhiane designed and performed the experiments and analyzed data. Marion Noulhiane manually delimited Regions of Interest. Charlotte Pinabiaux, Marion Noulhiane, Lucie Hertz-Pannier, Catherine Chiron, and Isabelle Jambaqué co-wrote the paper, discussed results, and commented on the manuscript.

### Conflict of interest statement

The authors declare that the research was conducted in the absence of any commercial or financial relationships that could be construed as a potential conflict of interest.

## Supplementary material

Figure S1 **Regions showing no subsequent memory effect (Misses > Hits) in medial temporal lobe during emotional face encoding**. Mean contrast values (Hits and Misses) by age group for fearful and neutral faces within ROIs in right **(A)** and left **(B**) medial temporal lobe (MTL). Bars represent mean square errors. Significant levels are indicated as follows: ^*^*p* < 0.01; ^***^*p* < 0.0005. Higher activations for Misses than for Hits for fearful faces were found in bilateral hippocampal heads and left hippocampal body, bilateral temporopolar and entorhinal cortices, right perirhinal cortex (*p*s < 0.00016), and left parahippocampal cortex (*p* = 0.0056) and for neutral faces in right entorhinal cortex (*p* < 0.00016) and hippocampal body (*p* = 0.00034). In 13–17 years group, bilateral parahippocampal cortex (right: *p* = 0.0062; left: *p* = 0.0048) and left hippocampal tail (*p* < 0.00016) displayed the Misses > Hits pattern for neutral faces but no regions were concerned for fearful faces.
