# Supplementary material for: Memory for fearful faces across development: specialization of amygdala nuclei and medial temporal lobe structures
Source: Front Hum Neurosci. 2013 Dec 25;7:901. doi: 10.3389/fnhum.2013.00901 (PMC3872298; doi:10.3389/fnhum.2013.00901)
Supplement: Figure S1 — Regions showing no subsequent memory effect (Misses > Hits) in medial temporal lobe during emotional face encoding. Mean contrast values (Hits and Misses) by age group for fearful and neutral faces within ROIs in right (A) and left (B) medial temporal lobe (MTL). Bars represent mean square errors. Significant levels are indicated as follows: *p < 0.01; ***p < 0.0005. Higher activations for Misses than for Hits for fearful faces were found in bilateral hippocampal heads and left hippocampal body, bilateral temporopolar and entorhinal cortices, right perirhinal cortex (ps < 0.00016), and left parahippocampal cortex (p = 0.0056) and for neutral faces in right entorhinal cortex (p < 0.00016) and hippocampal body (p = 0.00034). In 13–17 years group, bilateral parahippocampal cortex (right: p = 0.0062; left: p = 0.0048) and left hippocampal tail (p < 0.00016) displayed the Misses > Hits pattern for neutral faces but no regions were concerned for fearful faces. [file Presentation1.PDF]

Memory for fearful faces across development: specialization of amygdala nuclei and medial temporal lobe structures

(Supplementary Figure 1).

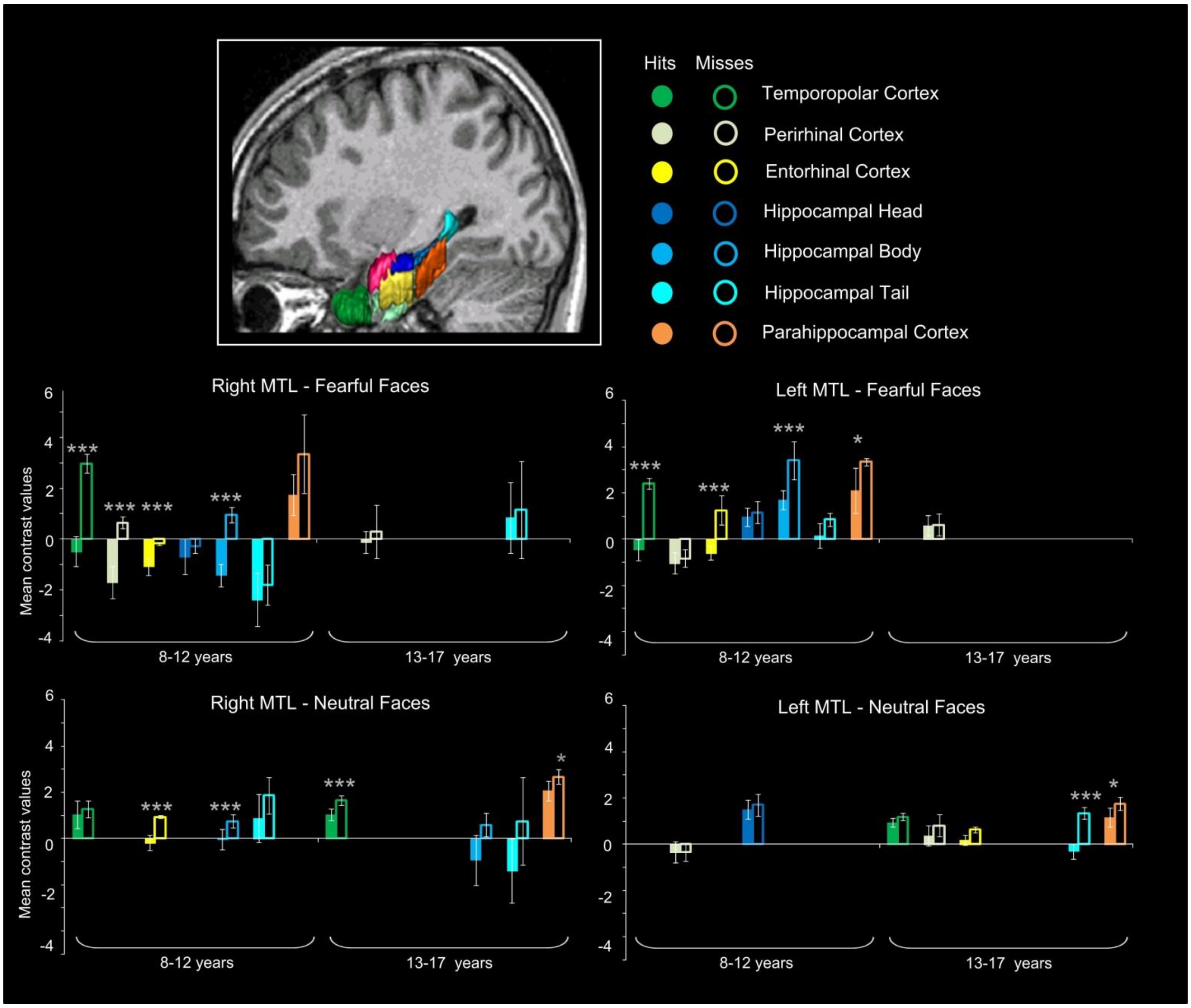

Regions showing no subsequent memory effect (Misses > Hits) in medial temporal lobe during emotional face encoding. Mean contrast values (Hits and Misses) by age group for fearful and neutral faces within ROIs in right (a) and left (b) medial temporal lobe (MTL). Bars represent mean square errors. Significant levels are indicated as follows: *p<.01; **p<.001; ***p<.0005. Higher activations for Misses than for Hits for fearful faces were found in bilateral hippocampal heads and left hippocampal body, bilateral temporopolar and entorhinal cortices, right perirhinal cortex (ps<.00016), and left parahippocampal cortex (p=.0056) and for neutral faces in right entorhinal cortex (p<.00016) and hippocampal body (p=.00034). In 13-17 years group, bilateral parahippocampal cortex (right: p=.0062; left: p=.0048) and left hippocampal tail (p<.00016) displayed the Misses > Hits pattern for neutral faces but no regions were concerned for fearful faces.
